# Temperature-associated selection linked to putative chromosomal inversions in king scallop (*Pecten maximus*)

**DOI:** 10.1098/rspb.2022.1573

**Published:** 2022-10-12

**Authors:** Christopher M. Hollenbeck, David S. Portnoy, Daniel Garcia de la serrana, Thorolf Magnesen, Iveta Matejusova, Ian A. Johnston

**Affiliations:** ^1^ Department of Life Sciences, Texas A&M University Corpus Christi, 6300 Ocean Drive, Corpus Christi, TX 78412, USA; ^2^ Texas A&M AgriLife Research, College Station, TX, USA; ^3^ Department of Cell Biology, Physiology and Immunology, Faculty of Biology, University of Barcelona, Barcelona, Spain; ^4^ Department of Biological Sciences, University of Bergen, Thormøhlensgt 53B, Bergen, Norway; ^5^ Marine Science Scotland, Marine Laboratory, 375 Victoria Road, Aberdeen AB11 9DB, UK; ^6^ Scottish Oceans Institute, School of Biology, University of St Andrews, St Andrews, Fife KY16 8LB, UK; ^7^ Xelect Ltd, Horizon House, Abbey Walk, St Andrews KY16 9LB, UK

**Keywords:** local adaptation, chromosomal inversion, population genomics, molluscs

## Abstract

The genomic landscape of divergence—the distribution of differences among populations or species across the genome—is increasingly characterized to understand the role that microevolutionary forces such as natural selection and recombination play in causing and maintaining genetic divergence. This line of inquiry has also revealed chromosome structure variation to be an important factor shaping the landscape of adaptive genetic variation. Owing to a high prevalence of chromosome structure variation and the strong pressure for local adaptation necessitated by their sessile nature, bivalve molluscs are an ideal taxon for exploring the relationship between chromosome structure variation and local adaptation. Here, we report a population genomic survey of king scallop (*Pecten maximus*) across its natural range in the northeastern Atlantic Ocean, using a recent chromosome-level genome assembly. We report the presence of at least three large (12–22 Mb), putative chromosomal inversions associated with sea surface temperature and whose frequencies are in contrast to neutral population structure. These results highlight a potentially large role for recombination-suppressing chromosomal inversions in local adaptation and suggest a hypothesis to explain the maintenance of differences in reproductive timing found at relatively small spatial scales across king scallop populations.

## 1. Introduction

The field of evolutionary genetics, driven by population genomic techniques, is increasingly concerned with the genomic landscape of divergence, which can be defined as the distribution of diversity across the genome within and among populations [1]. A common observation is the presence of ‘genomic islands of divergence’ among populations or species, which refers to genomic regions of high genetic differentiation flanked by regions of low differentiation [2,3]. Explanations for genomic islands of divergence initially focused on the interplay of selection and gene flow, hypothesizing that these regions contained variation important to local adaptation, thereby slowing the rate at which immigrant alleles move among populations, while gene flow homogenized allele frequencies in adjacent, selectively neutral regions [3,4]. However, recent research has demonstrated that genomic islands of divergence can arise under a variety of conditions, including scenarios without selection or gene flow [1,5,6].

Increasingly, chromosomal architecture is being implicated in the process of adaptation and formation of genomic islands of divergence [7,8], in large part because elements of chromosomal architecture including inversions, rearrangements and centromere location can reduce or prevent local recombination [9]. The effect is that alleles in regions of reduced recombination are frequently inherited as large units, amplifying the signals of forces that produce genomic islands across a larger genomic region. Chromosomal inversions, which often completely suppress recombination in inversion heterozygotes (however, see Navarro *et al*. [10]), may promote local adaptation through the maintenance of sets of co-adapted alleles at two or more loci (so-called ‘supergenes’; [11]). Chromosomal inversions have been implicated in driving differences in mating systems and local adaptation in a variety of taxa, including plants [12], birds [13,14], insects [15,16] and fishes [17–20].

Bivalve molluscs are a useful model system for investigating the relationship between genomic architecture and adaptation, as there is ample evidence of local adaptation across heterogeneous environments [21–23], as well as a growing body of evidence documenting an exceptional degree of genomic structural variation [24–26]. King scallop (*Pecten maximus*), also known as great scallop, is a high-value mollusc that supports a large fishery in the eastern North Atlantic ocean, and for which attempts to describe genetic population structure span decades [27–29]. The consensus among recent microsatellite and single nucleotide polymorphism (SNP)-based studies involving samples largely spanning the natural range of the species (Spain to Northern Norway) is the existence of an ‘Atlantic’ population (following the nomenclature of [30]) in the south (Spain to the UK) and a ‘Norwegian’ population in the north, with comparatively small differences observed among localities within these larger groups at neutral loci [30,31]. A recent study using restriction site-associated DNA sequencing (RADseq) was able to place the genetic discontinuity separating the two stocks in proximity to the Norwegian Trench, located between the Shetland Islands (UK) and Norway, and also reported the association of a subset of loci with environmental parameters, notably sea surface temperature, which tended to group individuals by latitude in contrast to the neutral structure [31].

Using a recent chromosome-level genome assembly [32], a population genomic survey of king scallop in the northeastern Atlantic Ocean was conducted to describe the genomic landscape of divergence in king scallops sampled from Galicia, Spain to north-central Norway, and a variety of genome scan and environmental association approaches were employed to assess population structure and genetic diversity at both neutral and putatively adaptive loci across the genome.

## 2. Methods

**Figure 1.**
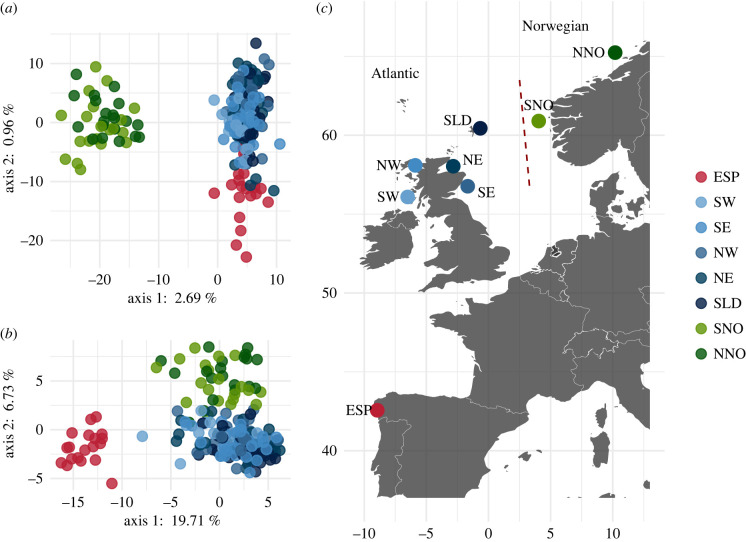
Study sampling distribution and neutral and outlier population structure. (*a*) Principal components analysis using 1852 neutral SNPs. (*b*) Principal components analysis using 68 SNPs identified as selection outliers by at least one test. (*c*) Map of samples collected in the current study: NNO, north Norway; SNO, south Norway; SLD, Shetland Islands; NE, northeast Scotland; SE, southeast Scotland; NW, northwest Scotland; SW, southwest Scotland; ESP, Spain. The red dashed line represents the approximate location of the Norwegian Trench. ‘Atlantic’ and ‘Norwegian’ refer to populations identified by previous population genetic analyses [30]. (Online version in colour.)

King scallops were sampled from eight localities in European waters of the eastern North Atlantic Ocean (figure 1). Individual king scallops from Scotland were sub-sampled from a larger set of individuals obtained from Marine Science Scotland survey trawls in 2015 and 2016 and included individuals from southwest (SW, *n* = 15), northwest (NW, *n* = 32), northeast (NE, *n* = 31) and southeast (SE, *n*= 29) Scotland, and the Shetland Islands (SLD, *n* = 35). Individuals from Norway were obtained from fish markets in the Hordaland (southern Norway; SNO, *n*= 20) and Trøndelag (north-central Norway; NNO, *n* = 20) regions and individuals from Galicia, Spain (ESP, *n* = 19) were obtained by diving. Further information, including details of sampling location, is presented in the electronic supplementary material, table S1.

Double-digest restriction-site-associated DNA libraries were prepared following Peterson *et al*. [33] for 225 unique individuals across the eight sample localities and were sequenced using 150 bp paired-end reads on two lanes of an Illumina HiSeq 4000 DNA sequencer. Raw sequence reads were demultiplexed with the program *process_radtags* from the Stacks (v. 1.47) software package [34]. Demultiplexed reads were processed with the *dDocent* (v. 2.6) pipeline [35], which performs quality trimming, read mapping and variant calling from the RAD data, and reads were mapped to a draft of the king scallop genome [32]. The resulting VCF file of genotypes was filtered stringently following O'Leary *et al*. [36] using the programs VCFtools v. 0.1.16 [37], vcflib v. 1.0.0-rc1 (https://github.com/vcflib/vcflib) and the R package *vcfR* v. 1.8.0 [38]. R scripts documenting the complete SNP filtering process can be found at https://www.github.com/chollenbeck/king_scallop_popgen_2022.

To facilitate linkage disequilibrium (LD) pruning and later haplotype-based tests for selection, the SNP genotypes were phased using the program Beagle [39,40]. SNPs in the resulting phased VCF file were then pruned for LD using the function *snp_autoSVD* in the R package *bigsnpR* [41]. This pruning step resulted in a ‘quasi-independent’ set of SNPs used for parameterizing the selection outlier tests, which is intended to eliminate or reduce bias caused by regions of low recombination [42].

Four genome scan methods were applied to identify loci potentially under the influence of natural selection: (i) a Bayesian differentiation outlier method implemented in the program Bayescan [43], (ii) a principal components analysis (PCA)-based differentiation outlier method implemented in the R package *pcadapt* [44], (iii) an environmental association method (latent factor mixed models; LFMM) implemented in the R package *LEA* [45], and (iv) an environmental association method (redundancy analysis; RDA) implemented in the R package *vegan* [46]. Environmental association analyses used sea surface temperature, extracted from a geographical grid of global monthly sea surface temperature data from January 1990 to December 2015 obtained from Ifremer's CORA dataset (available at http://www.ifremer.fr/erddap/griddap/CORA.html). Temperature values for each grid point were averaged across the entire time period to obtain a single estimate for each point on the grid, and the temperature estimate at the grid point nearest to the approximate geographical location of each sampling locality was used in the association analyses. In addition, phased genotypes were used to calculate two haplotype-based selection statistics: iES, a single-population measure of haplotype homozygosity (in this form the average length of shared haplotypes in a particular genomic region) indicative of positive selection [47,48], and Rsb, the log ratio of normalized iES between population pairs [49]. These methods were implemented in the R package *rehh* [50].

Results of the selection tests were used to separate the genotype data into two datasets: one containing loci that were identified as being putatively under directional selection by at least one of the genome scan methods and one containing the remainder of the putatively neutral loci. Population genetic structure was evaluated for both datasets separately using PCA, implemented in the R package *adegenet* [51,52]. Estimates of genetic diversity (expected and observed heterozygosity) for each sample locality and pairwise *F*_ST_ were calculated using *adegenet* and the R package *hierfstat* [53]. Pairwise *F*_ST_ was also estimated for sample localities grouped by region (Norway, Scotland and Spain), based on the results of the outlier PCA.

To further test for an association between sea surface temperature and genotype, allele frequencies for outlier loci in each locality were decomposed into a set of composite synthetic variables with correspondence analysis (CA), as implemented in *adegenet*. The first CA axis (corresponding to outlier PCA axis 1) was used as the dependent variable in a multiple linear regression with sea surface temperature as the independent variable and neutral genetic group (Norway versus Scotland/Spain) and latitude as covariates.

In order to test for the presence of putative chromosomal inversions or other regions of low recombination, pairwise LD for all loci within each locality and region (Norway, Scotland and Spain) was calculated using the R package *gaston* [54]. LD network analysis (LDna), as implemented in the R package *LDna* [55], was used to further explore the existence of chromosomal inversions on chromosomes 2, 8 and 12. First, a pairwise matrix of LD values was calculated with *gaston*, as above, with individuals at all localities grouped together. Single outlier clusters (SOCs) of loci linked together by LD were then identified, and the resulting LD network was visualized with *LDna* and the R package *ggnetwork* [56]. To explore the frequency of putative inversion genotypes, PCA was conducted, as above, but separately for SNPs contained within the boundaries, defined by LD blocks, of each putative inversion (local PCA; [57]). To identify putative inversion homozygotes and heterozygotes, the *find.clusters* function in *adegenet* was used to assign individuals to one of three clusters (presumably non-inverted homozygotes, inversion heterozygotes and inversion homozygotes). For each putative inversion, frequency of each inversion genotype, heterozygosity and conformance to Hardy–Weinberg equilibrium within individual localities were then calculated using *adegenet*.

Using the reference genome annotation, genes in the vicinity of each outlier region were extracted by selecting genes falling within 50 kb (25 kb upstream and downstream) of each outlier locus. In the case of large outlier clusters, all genes located within the bounds of the region were selected as candidate genes, whether or not they were within 50 kb of an outlier locus. Candidate genes were further refined by assigning outlier loci to two separate groups based on contribution to the principal components in the outlier PCA. Further details regarding methods used, including specific parameters, can be found in the electronic supplementary material, methods and in R scripts provided at https://www.github.com/chollenbeck/king_scallop_popgen_2022.

## 3. Results

The raw sequencing data contained 562.9 million read pairs, with a total of 514.5 million read pairs retained after demultiplexing. Following read mapping and variant calling, a total of 747 758 putative raw variants were discovered. Stringent filtering produced a set of 1920 SNPs that were used in all subsequent analyses.

**Figure 2.**
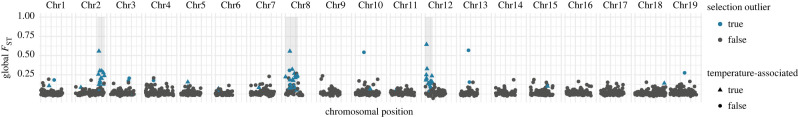
Global *F*_ST_ plotted against genomic position for all 19 *Pecten maximus* chromosomes. Blue points represent loci identified as being under the influence of natural selection by at least one test. Triangular points indicate loci significantly associated with sea surface temperature. Grey boxes highlight chromosomal regions spanning several megabases containing selection outliers. (Online version in colour.)

**Figure 3.**
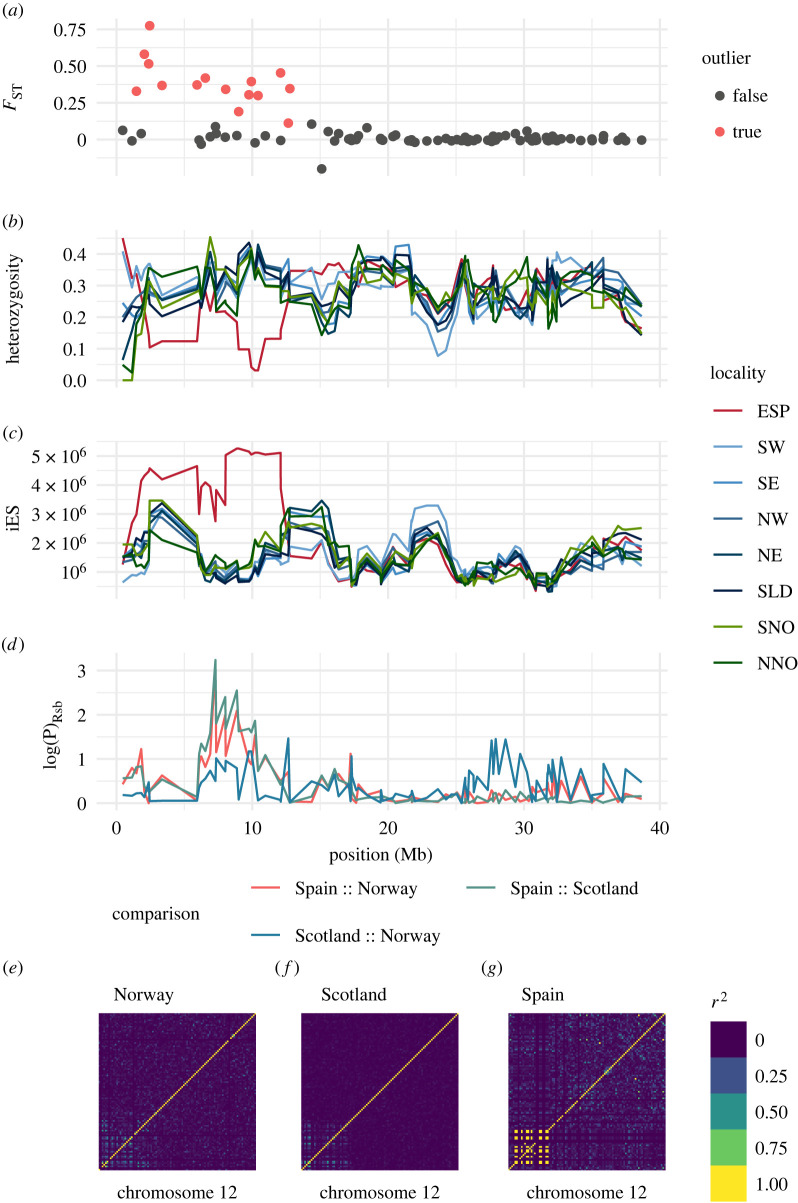
Signatures of selection at 97 SNPs on *Pecten maximus* chromosome 12. (*a*) Pairwise *F*_ST_ (Scotland/Spain) plotted against genomic position for chromosome 12; (*b*) smoothed expected heterozygosity plotted against genomic position for each locality; (*c*) iES, a statistic that measures the average length in base pairs of shared haplotypes (where larger values indicate larger regions of extended homozygosity, an indicator of a selective sweep) plotted against genomic position for chromosome 12; (*d*) log of the *p*-value for test of statistical significance of Rsb, the log-ratio of iES for pairs of populations, plotted against genomic position for chromosome 12; (*e,f,g*) heatmap of pairwise linkage disequilibrium (*r*^2^) for all loci on chromosome 12 for (*e*) Norwegian localities (*f*) Scottish localities and (*g*) Spain. Locality abbreviations: NNO, north Norway; SNO, south Norway; SLD, Shetland Islands; NE, northeast Scotland; SE, southeast Scotland; NW, northwest Scotland; SW, southwest Scotland; ESP, Spain. (Online version in colour.)

Sixty-eight loci putatively under the influence of selection were identified by at least one of the four methods. Eleven loci were identified with all four methods. Fifty-three loci were significantly associated with sea surface temperature, based on at least one environmental-association test, and 30 loci were significantly associated with both environmental-association methods. The genomic distribution of loci putatively under directional selection was non-random, with most loci grouped into one of three large regions (ranging from 12 to 22 Mb) on chromosomes 2, 8 and 12 (figure 2). The regions on chromosomes 2 and 12 exhibited high estimates of global *F*_ST,_ reduced heterozygosity, and increased levels of haplotype homozygosity (iES) in Spain (figure 3*a–d*; electronic supplementary material, figure S3A-D). The approximately 17 Mb region on chromosome 8 exhibited generally high estimates of pairwise *F*_ST_ and showed allele frequency differences similar to the other two regions but did not exhibit a reduction in heterozygosity or an increase in iES across the entire 17 Mb region in Spain (electronic supplementary material, figure S4A-D). For chromosome 8, the distribution of Rsb (indicative of directional or divergent selection) contained several smaller peaks rather than a single large peak.

PCA revealed that individuals grouped into three distinct ‘regional’ groupings: Norway, Scotland and Spain. The PCA involving only neutral loci revealed a primary component of variation (explaining 2.69% of the total variation) that differentiated Norway from Scotland and Spain, and a secondary component of variation (0.96% of the total) that differentiated Spain from Scotland and Norway (figure 1*a*). Estimates of pairwise *F*_ST_ based on the neutral dataset were at least three times larger for comparisons between localities in Norway and localities in Scotland or Spain (ranging from 0.036 to 0.045) than comparisons between localities in Scotland/Spain (ranging from 0.008 to 0.010). Fine-scale substructure was also detected between Scottish localities, with SW Scotland differing significantly at neutral loci (*F*_ST_ = 0.0043–0.0049) from NE Scotland and the Shetland Islands, and there was a small, but significant difference (*F*_ST_ = 0.005) between the two Norwegian localities (electronic supplementary material, table S2).

The PCA conducted using outlier loci revealed a contrasting pattern. The primary component of variation (19.5% of the total) differentiated Spain from Norway and Scotland, and the secondary component of variation (6.71% of the total) differentiated Norway from Scotland and Spain (figure 1*b*). SNPs contributing most to each outlier PC tended to group together in the genome, with loci contributing a larger effect to PC1 (the temperature/latitude-associated pattern; figure 1*b*, PC1; figure 2) tending to be located in the large regions identified on chromosomes 2, 8 and 12. The majority of loci that were significantly associated with sea temperature by the LFMM or RDA methods (41 of 53) fell into these regions. The SNPs contributing most to outlier PC2 (figure 1*b*, PC2) were located on chromosomes 3, 10, 13 and 19. A comparison of regional pairwise *F*_ST_ confirmed this pattern, showing that SNPs which strongly differentiated Spain from the other localities (high pairwise *F*_ST_) tended to be located in the same regions, while SNPs that differentiated Norway from the other localities also tended to group together in the genome (electronic supplementary material, figure S1).

The regression-based test for genotype-environment association with outlier loci was significant, both with sea surface temperature as the sole independent variable (adj. *R*^2^ = 0.877; *p* < 0.001) and after correcting for the effects of neutral genetic group and latitude (adj. *R*^2^ = 0.991; *p* < 0.001). Visualization of the CA and allele frequencies from the SNPs contributing most to CA axis 1 showed a north/south gradient in allele frequencies, with alleles in SW Scotland often intermediate to Spain and other Scottish localities (electronic supplementary material, figure S2).

Visualization of pairwise LD revealed that the three clusters of outlier loci on chromosomes 2, 8 and 12 fell into well-defined blocks of extended LD (figure 3*e*–*g*; electronic supplementary material, figures S3 and S4E-G), suggesting a reduction in recombination over a large segment of each of the chromosomes. For chromosome 12, LD was strongest in Spain, with a block of LD (*r*^2^ > 0.99) spanning at least 10 Mb (figure 3*g*). The same block of LD was apparent in chromosome 12 in Scotland and Norway, but at reduced levels of LD, as measured by *r*^2^ (figure 3*e*,*f*). For chromosome 2, the LD block was more apparent in Scotland and Norway, but largely because it was not possible to measure LD in Spain owing to fixation of many of the SNPs in the LD block. The LD block on chromosome 8 was largest (greater than 17 Mb) and most clearly defined in Norway (electronic supplementary material, figure S4E), although elevated LD could still be seen in the same chromosomal region in Scotland (electronic supplementary material, figure S4F), and LD was not able to be estimated at all loci owing to fixation of several loci in Spain. LDna identified five SOCs containing more than three loci on chromosomes 2 (19 loci; chr2: 43819604–55023173, 8 (six loci; chr8: 8584060–22490951) and 12 (10 loci; chr12: 1468734–12766088) (electronic supplementary material, figure S5 and table S3). Three of these SOCs (one on each chromosome) corresponded to regions containing LD blocks identified with previous analyses. In addition, two overlapping SOCs with relatively lower median *r^2^* (containing four and six loci) were identified adjacent to the major SOC on chromosome 2.

**Figure 4.**
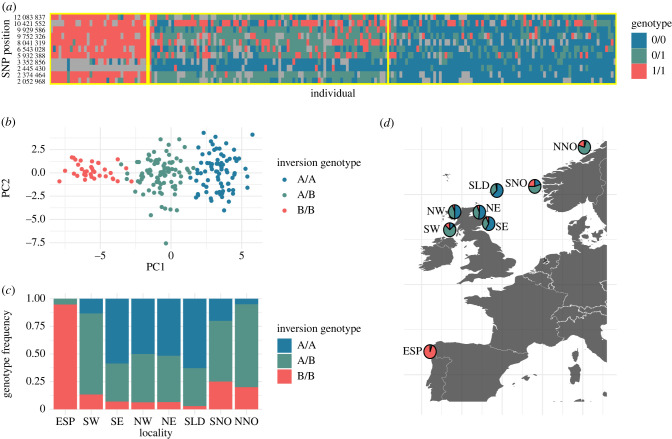
Population frequencies of inversion genotypes on chromosome 12. (*a*) Genotype heatmap of all individuals (*x*-axis) at SNPs contained within the putative inversion on chromosome 12 (*y*-axis). Colours represent SNP genotypes (blue, homozygote; green, heterozygote; red, alternate homozygote; grey, missing genotype) and yellow boxes indicate genotype clusters in (*b*). (*b*) Local PCA of putative inversion on chromosome 12 showing clusters of inversion genotypes. (*c*) Population frequencies of inversion genotypes. (*d*) Sample map with inversion genotype frequencies. (Online version in colour.)

In general, SNP loci within the three LD blocks showed similar patterns of allele frequency differences among localities (electronic supplementary material, figure S2C), but the frequency of putative inversion genotypes across localities differed among the three LD blocks. For chromosomes 2 and 12, local PCA grouped individuals into three genotype clusters (figure 4; electronic supplementary material, figure S6). For chromosome 2, putative inversion genotypes did not deviate from the expectations of Hardy–Weinberg equilibrium in all localities and allele frequencies were similar in Scotland and Norway, with one inversion allele being completely fixed in Spain (electronic supplementary material, figure S6). For chromosome 12, one putative inversion allele that was nearly fixed in Spain was found in intermediate frequencies in Norway and at lower frequencies in Scotland. In addition, an excess of heterozygotes (*α* = 0.1) was found in northern Norway (NNO, figure 4; *p* = 0.016) and also in the SW Scotland locality (SW, figure 4; *p* = 0.070). The LD block on chromosome 8 revealed a more complex pattern of divergence than the putative inversions on chromosomes 2 and 12, with a component of variation differentiating Spain from the other localities and a component where Norway was differentiated from all other localities (electronic supplementary material, figure S7).

A total of 2840 genes were identified to be in proximity to an outlier SNP or contained within one of the three LD blocks. Of these, 2774 were in proximity to SNPs contributing a larger effect to outlier PC1 (latitudinal effect), while 66 genes were in close proximity to genes contributing more to outlier PC2 (differentiating Norway from Atlantic localities).

## 4. Discussion

A chromosome-level reference genome and a genotyping-by-sequencing approach was used to explore the genomic landscape of divergence in king scallop in the NE Atlantic. Neutral population genetic structure was in concordance with the results of previous studies [30,31], supporting the existence of distinct Atlantic (Spain to the UK) and Norwegian populations, with a genetic discontinuity occurring in the proximity of the Norwegian Trench separating the Shetland Islands, Scotland, from Norway. Smaller scale neutral genetic differences were also observed within the two larger populations, but a more complete sampling is needed to resolve whether these differences represent distinct local subpopulations [58] or whether an isolation by distance effect is present. Putatively adaptive genetic variation revealed two patterns of structure, with each pattern being driven by loci localized to separate regions of the genome. The first, minor pattern was spatially congruent with the neutral pattern of variation (distinguishing Atlantic and Norwegian groups) but driven by outlier loci with large differences between Norwegian and Atlantic groups and may reflect regions of the genome involved in local adaptation related to larger-scale regional (Atlantic versus Norwegian) conditions. The second, more pronounced pattern of adaptive genetic variation observed, as summarized by outlier PC1, was characterized by a latitude-associated pattern in which the southernmost locality, Spain, showed a high degree of divergence in allele frequencies from other localities. This component of variation was significantly associated with sea temperature and was almost entirely driven by loci localized to three large LD blocks on chromosomes 2, 8 and 12.

### (a) Evidence for chromosomal inversions

While localized reduction in recombination can be caused by several possible aspects of chromosome architecture, including proximity to centromeres [59] and structural variation (particularly chromosomal rearrangements such as inversions; [9]), two pieces of evidence suggest that the three large outlier regions and blocks of LD identified correspond to chromosomal inversions. First, the LD blocks identified are all relatively large (approx. 11–15 Mb) with well-defined boundaries, suggesting the presence of inversion breakpoints. As an example, on chromosome 12, LD in Spain drops sharply from nearly 1 to background levels (less than 0.01) moving between adjacent SNPs that span the boundary of the LD block between the SNPs at positions 12 083 837 and 12 091 012. Second, in certain localities increased LD can be seen between loci flanking either side of the block of LD (figure 3*e*), which would occur if these loci are adjacent in particular inversion genotypes or if there are multiple small inversions present. The hypothesis of the existence of inversions can be tested in future studies using alternative methods to those presented here, including genetic mapping [60], whole-genome resequencing [20] or polymerase chain reaction-based methods [61].

Chromosomal inversions linked to adaptation have recently been described in several marine systems, notably in Atlantic cod [17,62] and threespine sticklebacks [63], as well as in the rough periwinkle, an intertidal marine snail [60]. The presence of chromosomal inversions is concordant with recent findings that genomic structural variation may be extremely prevalent in bivalve molluscs. Calcino *et al*. [24] recently showed using highly contiguous reference genome assemblies from several molluscan species that individual bivalves tended to be hemizygous at approximately 4–7 per cent of the genome, with the king scallop genome showing 6.14% hemizygosity. In addition, a high-quality assembly of the Mediterranean mussel genome and resequencing of 14 individuals [25] revealed that approximately 25% of genes were found to be missing owing to the presence–absence variation in at least one of the resequenced individuals. The authors of these studies have suggested that widespread genomic structural variation in molluscs may support an ability to rapidly adapt to heterogeneous environmental conditions despite a high degree of connectivity, a hypothesis further supported by the results of this study.

### (b) Adaptive genetic variation

One key result of the present study that highlights the benefits of establishing a genomic position for loci in a population genomics context is the finding that patterns of divergence revealed by each of the two primary outlier PCs tended to be driven by loci grouped in separate regions of the genome. The loci contributing to the weaker, secondary pattern of adaptive genetic variation (spatially congruent with neutral structure) were found as singletons or pairs of outlier SNPs on chromosomes 3, 10, 13 and 19, and potentially represent loci that promote local adaptation across larger regional populations (Norwegian and Atlantic). However, the fact that allele frequency patterns within these loci are congruent with the overall neutral signal make it difficult to rule out the effects of purely demographic processes [64]. One observation that supports the effects of selection rather than drift alone is the presence of related genes in multiple areas of the genome exhibiting the same signal: five of the 66 genes identified within 50 kb of these outlier loci (a tandem array of three genes on chromosome 19 and an array of two genes on chromosome 10) were serine/threonine kinases, a family of proteins that have been observed to be highly upregulated in scallop gonads [65,66]. Phenotypic differences between Norwegian and Atlantic king scallops have been documented involving growth rates [67] and in proteomic comparisons [68], but further work is needed to determine the underlying mechanisms behind these between-region differences.

The second observed pattern of adaptive genetic variation involved significant differences in frequencies of temperature-associated alleles within putative inversions, which was in contrast to neutral population structure. Reduction in heterozygosity and elevated iES for putative inversions located on chromosomes 2 and 12 observed in Spain are evidence for strong positive selection (i.e. selective sweeps) in these genomic regions [48]. Significant heterozygote excess for the putative inversion on chromosome 12 observed in Norway and SW Scotland suggests that balancing selection may also have an important role in shaping inversion allele frequencies. While inversion heterozygotes may incur a fitness cost owing to the inviability of gametes when recombination occurs within inverted regions, selection for inversion heterozygotes genotypes via overdominance, frequency-dependent selection, or selection in spatially/temporally heterogeneous environments can overcome this barrier [8]. A well-documented example of heterogeneous selection favouring chromosomal inversions is seen in *Drosophila melanogaster*, where allele frequencies in some populations have been shown to fluctuate seasonally in response to variables such as temperature, independently of neutral population structure [16]. Balancing selection associated with inversion polymorphisms in response to heterogeneous environmental conditions has also been described in related *Drosophila subobscura* [69] and *Anopheles* mosquitos [70]. Further evidence for spatio-temporally heterogeneous selection in this study is the observation that SW Scotland, which has warmer sea temperatures compared to the other northern localities sampled here, exhibited heterozygote excess, suggesting that putative inversion heterozygotes may be favoured at intermediate latitudes within the Atlantic population. The excess of putative inversion heterozygotes in north-central Norway is also consistent with this, as these individuals were sampled in an area intermediate to groups shown to have different life-history characteristics in the south and north of Norway [71]. However, one limitation of the current study is a lack of spatially continuous sampling along the European coast, which would help to further elucidate whether the patterns detected here are clinal in nature or spatially discrete, as well as defining the spatial scales at which local adaptation is important.

### (c) Structural variation, genomic islands and adaptation

Genomic islands of divergence are hypothesized to arise under a variety of conditions, and these mechanisms can be broadly classified by whether zero (selection is not involved), one, or multiple loci within an island are targets of selection [6]. Chromosomal rearrangements, by producing regions of low recombination, can be involved in any of these scenarios. In the case of king scallop, it is unlikely that purely demographic processes (e.g. allele surfing owing to range expansion; [72]) are responsible for the observed signal, based on the fact that the primary outlier signal contrasts with neutral structure. Without further data, it is difficult to distinguish mechanisms that involve a single target of selection, for example genetic hitchhiking associated with directional or divergent selection acting upon a single locus [73] or background selection against deleterious alleles [74], from multi-locus mechanisms, such as co-adapted gene complexes or ‘supergenes’ [11]. The physical size of the putative inversions (approx. 11–15 Mb) and the existence of multiple peaks of reduced diversity and/or increased divergence within individual islands (figure 3; electronic supplementary material, figures S3 and S4) observed here, are potential evidence for selection acting on multiple loci within each large region [6].

Connecting the genomic landscape revealed here to adaptive mechanisms will require further work; however, one hypothesis relates to the fact that temperature is known to be among the most important factors influencing timing of gametogenesis and spawning in bivalve molluscs, including *P. maximus* [75,76]. Previous studies have reported the major spawning period for *P. maximus* to be roughly May through to August [77], with some studies reporting additional peaks in spawning activity in autumn or winter, notably in Spanish populations [78]. It has also been noted that natural populations tend to exhibit one of two different reproductive cycles—one in which individuals rebuild gonads quickly after spawning and another in which individuals wait until the next year to rebuild gonads [79], and populations exhibiting these different reproductive tendencies have been identified at relatively small spatial scales within the Norwegian [71] and Atlantic [80,81] populations. Further, transplant studies have demonstrated that individuals relocated early in life to areas with different reproductive cycles maintain the reproductive cycle of their source population in the new environment, suggesting heritable differences in the timing of reproductive development [71,81,82]. However, few differences in neutral genetic variation have been identified at the same geographical scales [27,30,31], consistent with the high potential for dispersal owing to a relatively long pelagic larval duration (18–42 days; [75]. The evidence for an association between temperature and specific components of genetic variation, originally reported by Vendrami *et al*. [31], combined with the evidence that temperature-associated genetic variation is associated with putative chromosomal inversions reported here, supports the hypothesis that differences in the optimal timing of reproductive development and spawning may be driven by localized selection operating on suites of co-adapted genes contained within chromosomal inversions that allow for the maintenance of locally adapted variation despite the high potential for connectivity at small spatial scales. Inversion-mediated adaptive divergence in the face of high potential for gene flow is exemplified in the well-characterized gastropod *Littorina saxatilis*, in which ecotypes characterized by differences in the frequencies of inversion polymorphisms have been observed along intertidal transects on the scale of tens of metres [60,83]. While the divergent phenotypes explored to date in the *Littorina* system have been largely morphological, similar observations have been made involving Atlantic cod and Pacific herring, where genetic variation associated with reproductive timing and strategy has also been associated with putative chromosomal inversions [17,84].

The size of the LD blocks and the large number of genes contained within them make it difficult to identify specific candidate genes that may be the targets of selection, particularly because of the possibility that a single gene under selection within the inversion could influence allele frequencies within the entire region. However, a number of genes previously linked to gonad-specific expression in scallops are present in the putative inversions. These include several serine/threonine protein kinases and phosphatases (two tandem serine/threonine kinases on chromosome 2 and four serine/threonine protein phosphatases on chromosome 12) and two adenosine deaminase-like genes on chromosome 8, all of which have been shown to be differentially expressed in male and female scallop gonads [65,66]. In addition, multiple genes related to serotonin transport and signalling were found in the putatively inverted regions (two 5-hydroxytryptamine receptor-like genes on chromosome 12 and one sodium-dependent serotonin transporter-like gene on chromosome 2). Serotonin is known to be intimately involved in the process of oocyte maturation in scallops and other bivalves [85], and is known to be an effective inducer of spawning in many bivalve molluscs [86]. Overall, the suite of inversion-associated genes identified here will be a rich set of candidate genes for future studies to attempt to identify the targets of selection associated with these regions.

## 5. Conclusion

Observing and understanding the genomic landscape of divergence, which is now possible owing to ever-improving genome sequencing and assembly techniques, allows for a more sophisticated view of microevolutionary processes because it incorporates the effects of local recombination, in addition to migration, drift and selection. The results presented here demonstrate an association between sea temperature and genetic variation in specific regions of the genome characterized by local reductions in recombination and highlight the importance of establishing genomic context in disentangling the effects of microevolutionary forces. These results suggest a mechanism by which broadcast spawning species with a high degree of connectivity can maintain genetic differences that allow for local adaptation. Further work in king scallops and other taxa to better characterize these systems will help to improve our understanding of how chromosome structure variation contributes to evolutionary change.

## Data Availability

Data and code (Rmd files) necessary for reproducing the results of the study can be found at https://www.github.com/chollenbeck/king_scallop_popgen_2022. Raw DNA sequence data can be found at the NCBI Short Read Archive (SRA) under BioProject Accession PRJEB20627. Additional raw data files (unfiltered SNP data) can be found on Dryad: https://dx.doi.org/10.5061/dryad.ttdz08m26 [87]. Data are provided in the electronic supplementary material [88].
